# Metaverse Clinic for Pregnant Women With Subclinical Hypothyroidism: Prospective Randomized Study

**DOI:** 10.2196/64634

**Published:** 2025-02-05

**Authors:** Yuanyuan Zheng, Yizhen Chen, Yan Chen, Liang Lin, Ting Xue, Chuhui Chen, Junping Wen, Wei Lin, Gang Chen

**Affiliations:** 1 Shengli Clinical Medical College Fujian Medical University Fuzhou China; 2 Department of Geriatric Medicine Fujian Key Laboratory of Geriatrics Diseases, Fujian Provincial Center for Geriatrics Fuzhou University Affiliated Provincial Hospital Fuzhou China; 3 Department of Endocrinology Fuzhou University Affiliated Provincial Hospital Fuzhou China; 4 Department of Obstetrics and Gynaecology Fuzhou University Affiliated Provincial Hospital Fuzhou China; 5 Department of Health Management Fuzhou University Affiliated Provincial Hospital Fuzhou China

**Keywords:** metaverse, subclinical hypothyroidism, adverse pregnancy outcomes, psychological health, neurobehavioral development

## Abstract

**Background:**

Health care is experiencing new opportunities in the emerging digital landscape. The metaverse, a shared virtual space, integrates technologies such as augmented reality, virtual reality, blockchain, and artificial intelligence. It allows users to interact with immersive digital worlds, connect with others, and explore unknowns. While the metaverse is gaining traction across various medical disciplines, its application in thyroid diseases remains unexplored. Subclinical hypothyroidism (SCH) is the most common thyroid disorder during pregnancy and is frequently associated with adverse pregnancy outcomes.

**Objective:**

This study aims to evaluate the safety and effectiveness of a metaverse platform in managing SCH during pregnancy.

**Methods:**

A randomized controlled trial was conducted at Fujian Provincial Hospital, China, from July 2022 to December 2023. A total of 60 pregnant women diagnosed with SCH were randomly assigned into two groups: the standard group (n=30) and the metaverse group (n=30). Both groups received levothyroxine sodium tablets. Additionally, participants in the metaverse group had access to the metaverse virtual medical consultations and metaverse-based medical games. The primary outcomes were adverse maternal and offspring outcomes, and the secondary outcomes included the neurobehavioral development of offspring and maternal psychological assessments.

**Results:**

Of the 30 participants in each group, adverse maternal outcomes were observed in 43% (n=13) of the standard group and 37% (n=11) of the metaverse group (*P*=.60). The incidence of adverse offspring outcomes was 33% (n=10) in the standard group, compared to 7% (n=2) in the metaverse group (*P*=.01). The Gesell Development Scale did not show significant differences between the two groups. Notably, the metaverse group demonstrated significantly improved scores on the Self-Rating Depression Scale and the Self-Rating Anxiety Scale scores compared to the standard group (*P*<.001 and *P*=.001, respectively).

**Conclusions:**

The use of metaverse technology significantly reduced the incidence of adverse offspring outcomes and positively impacted maternal mental health. Maternal adverse outcomes and offspring neurobehavioral development were comparable between the two groups.

**Trial Registration:**

Chinese Clinical Trial Registry ChiCTR2300076803; https://www.chictr.org.cn/showproj.html?proj=205905

## Introduction

The metaverse is a shared virtual space that seamlessly blends the physical and digital realities. It offers an immersive environment where users can interact in a computer-generated universe while gaining real-world benefits [1]. Built upon the foundations of mixed reality, artificial intelligence, and blockchain technology, the metaverse leverages these cutting-edge innovations to deliver dynamic, interactive, and secure user experiences [1-3]. Mixed reality, which includes both augmented reality and virtual reality (VR), bridges the continuum between physical and virtual experiences. Artificial intelligence enables adaptive and intelligent environments, while blockchain technology ensures secure transactions and ownership of digital assets, thereby supporting a resilient digital economy within the metaverse. In health care, the metaverse holds transformative possibilities [4,5]. Compared to traditional health care models, it enables secure and customizable therapeutic environments tailored to the unique needs and treatment goals of individual patients. By integrating the virtual and physical realms, it also allows for real-time monitoring and feedback [1,6]. By 2030, the global metaverse health care market is projected to reach US $98.3 billion, with the United States expected to lead this growth, followed by Europe and the Asia-Pacific region [2]. Early applications of metaverse technologies in health care have primarily focused on surgery, particularly in urology, neurosurgery, and surgical training [7-9]. Recently, its applications have expanded to areas such as cognitive rehabilitation, mental health, and chronic disease management [1,2,10]. Despite these promising developments, its potential role in the management of thyroid disorders remains unexplored.

Subclinical hypothyroidism (SCH), characterized by elevated thyrotropin levels with normal free thyroxine (FT4), is increasingly prevalent during pregnancy [11,12]. SCH is associated with adverse pregnancy outcomes, including spontaneous abortion, preeclampsia, and placental abruption [11,13]. Timely treatment with levothyroxine (LT4) sodium tablets is critical but is often hindered by limited awareness and delayed diagnosis [14].

Aligned with the Institute of Medicine’s framework for high-quality health care—which prioritizes patient-centeredness, equity, safety, effectiveness, timeliness, and efficiency—our team has developed an innovative metaverse-based platform tailored for pregnant women with SCH [15]. This digital solution provides personalized care through comprehensive follow-up, starting from the antenatal period and extending to 3 months post partum. The platform aims to enhance SCH management by enabling timely and effective interventions, optimizing resource use, and promoting long-term maternal health. Our primary goal is to assess the safety and effectiveness of this metaverse-based platform in managing SCH during pregnancy. By addressing unmet clinical needs, the initiative seeks to reduce complications and improve the overall quality of prenatal and postnatal care.

## Methods

### Ethics Approval

This single-center, randomized, open-label trial was approved by the Fujian Provincial Hospital Institutional Review Board (K2022-06-016) and the research ethics board. The trial was registered under ChiCTR2300076803. Recruitment, treatment, and follow-up were conducted from July 2022 to December 2023.

Participants in this clinical trial received compensation for their time and involvement. Compensation was provided to recognize the participants’ contributions and to help offset any expenses associated with their participation. Participants who attend the screening visit will receive compensation for their time and travel expenses. Participants will be compensated for each study visit attended. This includes time spent on medical assessments, laboratory tests, and any other required procedures (¥500-600, US $70-$80). Any out-of-pocket expenses incurred during participation, such as transportation or childcare, may be reimbursed upon submission of receipts. Upon successful completion of all study visits, participants will receive a completion bonus (¥1000, US $150).

### Participants

Study participants were conveniently recruited from the Fujian Provincial Hospital outpatient practice and prescreened during appointments scheduled for SCH evaluations. Eligibility was confirmed through in-person assessments to ensure compliance with inclusion criteria. Written informed consent was obtained from all participants, and all data were anonymized or deidentified to protect confidentiality.

The inclusion criteria were as follows: pregnant women diagnosed with SCH, as defined by the 2017 American Thyroid Association Guidelines for the Diagnosis and Management of Thyroid Disease During Pregnancy and Postpartum (serum thyrotropin levels above the pregnancy-specific upper reference limit, or ≥4.0 mIU/L, with normal FT4 levels [16]), diagnosis and initiation of LT4 treatment before 8 weeks of gestation, singleton pregnancy, and age ≥18 years. The exclusion criteria were a history of thyroid disease; history of residence in areas with endemic goiter; use of LT4 before pregnancy or medications that affect thyroid function; history of nonthyroidal autoimmune diseases; preexisting cardiovascular, liver, or kidney diseases; or any prepregnancy condition that could interfere with the study. A total of 60 participants were enrolled in the trial.

### Randomization and Masking

Participants were randomly assigned in a 1:1 ratio to either the standard or metaverse group using computer-generated randomization. Both groups were managed by the same medical team. Investigators and participants were not masked to treatment allocation. The research team, data manager, and statistician were aware of group assignments during the process of data collection. Data were securely stored in the Fujian Provincial Hospital database, with the first author overseeing data accuracy and integrity. No placebo was used in the study. As predefined guidelines exist for LT4 use, the potential bias introduced by the nonblinded design on the primary outcome was considered minimal.

### Procedures

The research team comprised a chief endocrinologist, an attending endocrinologist, an obstetrician-gynecologist, and a physician specializing in health management, all with extensive experience in thyroid disorder management. Eligible patients were prescribed oral LT4 (Euthyrox, Berlin-Chemie AG, Germany; H20110178, 50 μg × 100 tablets) based on thyroid function test results [16]. Initial dosages ranged from 25 to 50 μg and were adjusted according to pregnancy status [16]. Patients took one tablet daily on an empty stomach in the morning, with thyrotropin and FT4 levels monitored every 14 days for dose adjustments. Once thyrotropin levels were within 0.1-2.5 mIU/L, monitoring was conducted every 4 weeks, with doses adjusted to maintain trimester-specific target ranges [16]. Treatment continued consistently until delivery.

Participants in the standard group attended regular in-person follow-ups to monitor blood pressure, heart rate, weight, thyroid function, medication dosage, and other pregnancy-related indicators. Participants in the metaverse group only visited the hospital for enrollment, blood tests, and required pregnancy assessments. Follow-ups for the metaverse group were conducted through the platform, with medications delivered to their homes. The metaverse platform enabled secure communication between participants and investigators at any time, enhancing adherence to treatment protocols. Technical support was provided, with hardware promptly replaced if issues could not be resolved remotely. Participants used home monitoring devices under investigator guidance, with training provided for platform use. Weekly assessments ensured proficiency. Participants identified as “unfamiliar” received additional training until they could fully engage in medical activities on the platform. The platform supported uploading and recording thyroid function test results and other relevant health data. It also offered lifestyle guidance, psychological counseling, and educational games focused on endocrinology. Immersive experiences using Pico VR glasses (Fujian Huayu Education Technology Co Ltd, China) provided gamified learning and live discussions with physicians. The standard group participated in similar educational activities during weekly in-person sessions.

Adverse pregnancy outcomes were tracked through follow-ups and patient self-reports. Offspring neurobehavioral development was assessed using Gesell Development Scale (GDS) scores at 1 and 3 months of age, evaluating gross motor, fine motor, adaptability, language, and personal-social skills. Maternal mental health was assessed using the Self-Rating Anxiety Scale (SAS) and Self-Rating Depression Scale (SDS) at baseline, during the last trimester, and 6 weeks post partum. Psychological support was offered based on the SAS/SDS results, with interventions delivered via the metaverse platform for the metaverse group and through outpatient sessions for the standard group.

The intervention spanned from pregnancy through 3 months post partum, with participation continuing unless interrupted by withdrawal or loss to follow-up.

### Outcomes

The primary outcomes of the study focused on assessing adverse maternal and offspring outcomes. Maternal adverse outcomes included gestational diabetes, gestational hypertension, premature rupture of membranes, amniotic fluid abnormalities, postpartum hemorrhage, and related complications [17]. Offspring adverse outcomes included fetal distress, abortion, stillbirth, preterm birth, neonatal asphyxia, and related complications [13,17]. Secondary outcomes focused on offspring neurobehavioral development and maternal mental health. Neurobehavioral development was considered normal if the developmental quotient on the GDS exceeded 85 [18]. Anxiety was defined as SAS scores greater than 50, and depression was defined as SDS scores greater than 53 [19]. Investigators conducted GDS assessments, while participants completed SAS and SDS questionnaires independently, which were collected immediately after completion.

### Statistical Analysis

Based on a significance level of 5%, statistical power of 90%, and an anticipated 5% loss to follow-up, a total of 60 participants (30 per group) was calculated as sufficient for this randomized controlled trial.

Quantitative data were expressed as means and SDs, while categorical data were presented as percentages. Between-group comparisons of normally distributed variables used the independent samples 2-tailed *t* test or Mann-Whitney *U* test, while categorical variables were analyzed using Pearson *χ*^2^ test or Fisher exact test. Analysis of covariance, with baseline thyroid function and initial psychological scores as covariates, was performed to assess postintervention differences in thyroid function and psychological outcomes between groups. Statistical analyses were conducted using SPSS 25.0 (IBM Corp), with a *P* value <.05 considered statistically significant.

## Results

### Overview

**Figure 1:**
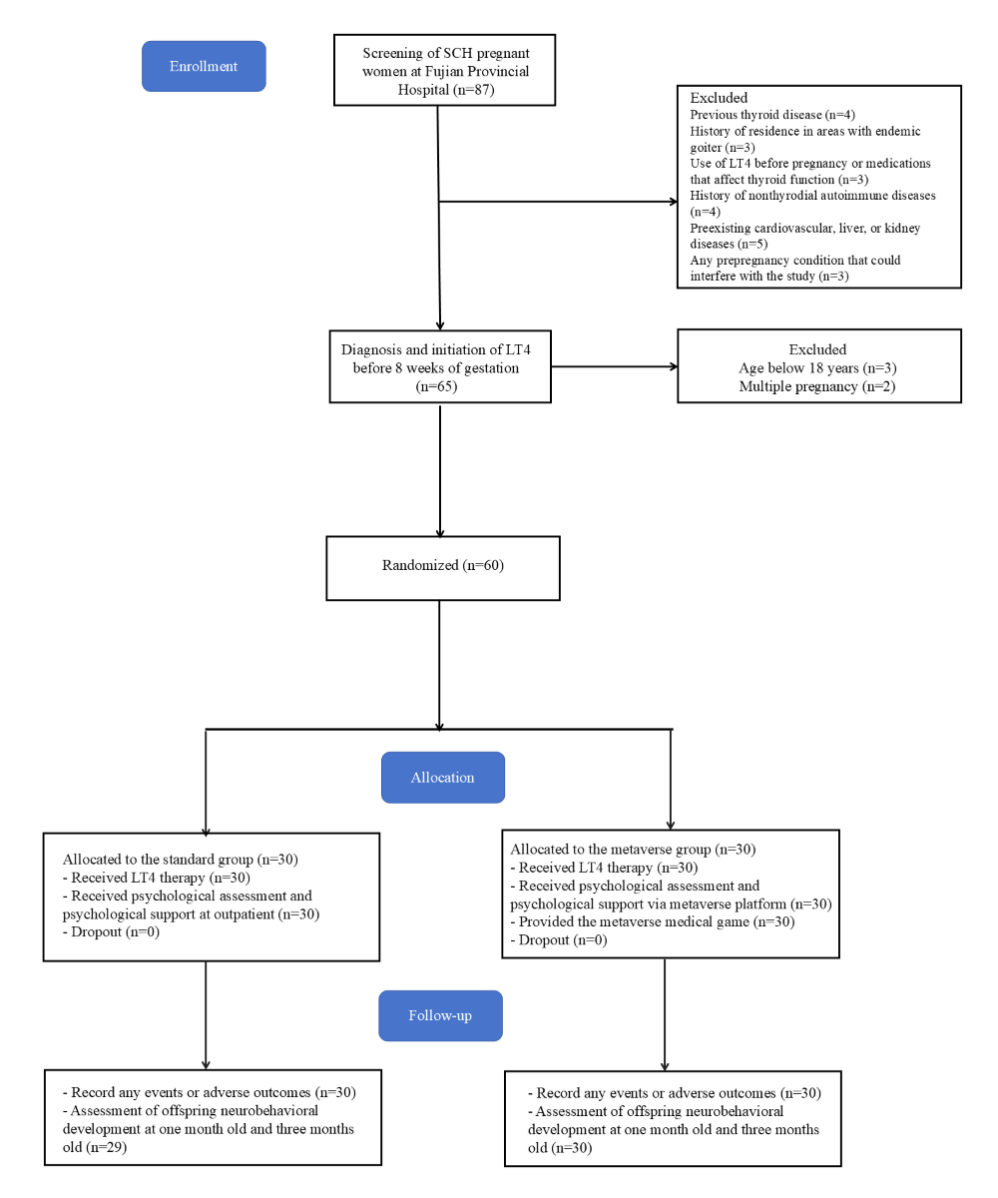
CONSORT (Consolidated Standards of Reporting Trials) flow diagram shows the number of participants. LT4: levothyroxine; SCH: subclinical hypothyroidism.

**Figure 2:**
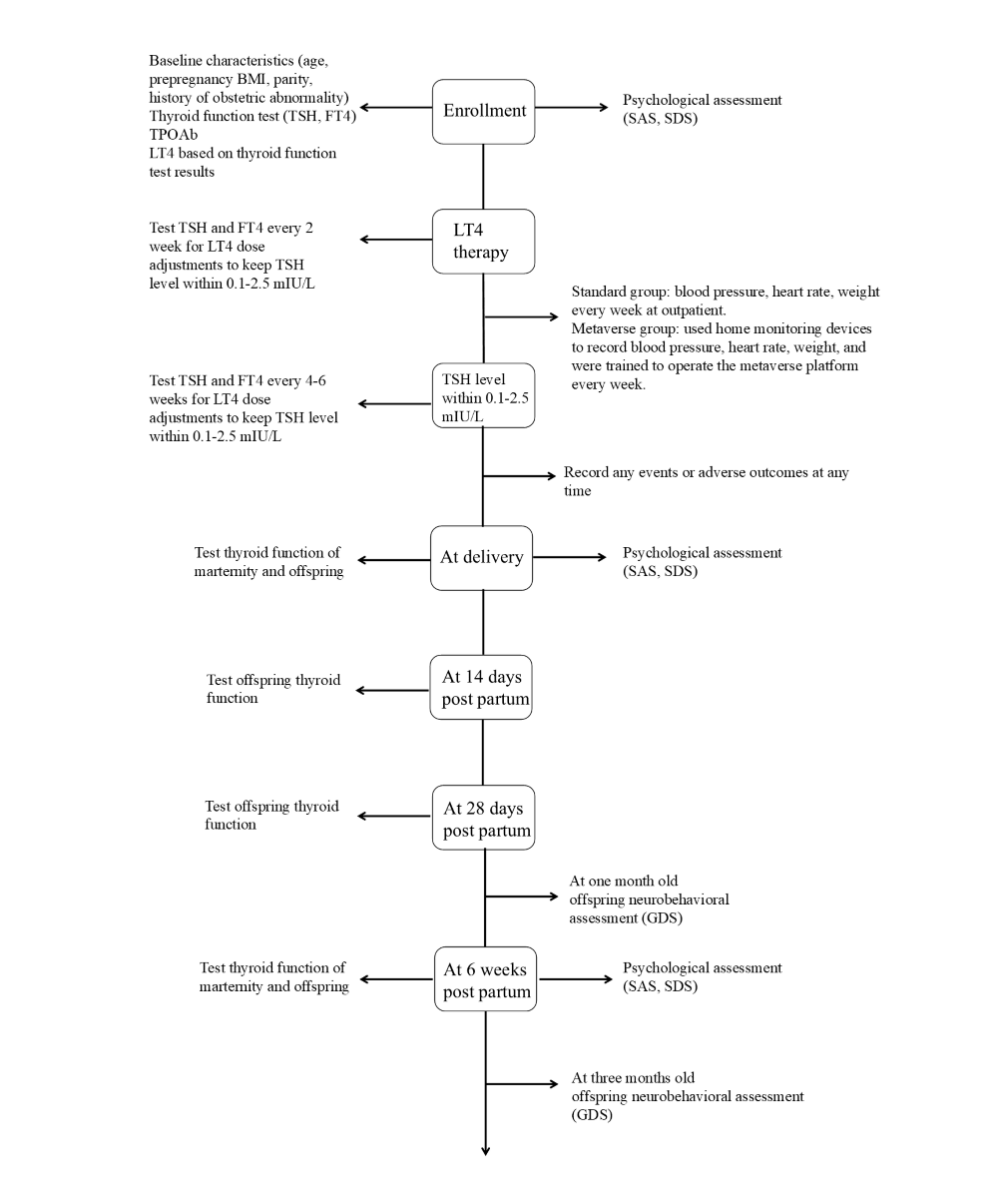
The timeline of the study and assessments. FT4: free thyroxine; GDS: Gesell Development Scale; LT4: levothyroxine; SAS: Self-Rating Anxiety Scale; SDS: Self-Rating Depression Scale; TPOAb: thyroid peroxidase antibodies; TSH: thyrotropin.

Between July 2022 and July 2023, 87 individuals were screened for eligibility, with 60 participants randomized equally into the standard group (n=30) and the metaverse group (n=30; Figure 1). By December 2023, all participants had completed the trial, including follow-up through 3 months post partum (Figure 2).

The median age of participants was 30 years in both groups, with age ranges of 20-40 years in the standard group and 23-38 years in the metaverse group. The median gestational age at delivery was 39 weeks, ranging from 23 to 40 weeks in the standard group and 36 to 40 weeks in the metaverse group. The median prepregnancy BMI was 22.8 (IQR 15.8-32.7) kg/m^2^ in the standard group and 21.5 (IQR 17.6-30) in the metaverse group. There were 19 (63%) nulliparous participants in the standard group and 18 (60%) in the metaverse group. Three participants in each group had a history of obstetric abnormalities (Table 1).

**Table 1:** Baseline characteristics of study participants (n=60).

		Standard group (n=30)	Metaverse group (n=30)
Age (years), median (IQR)		30 (20-40)	30 (23-38)
Gestational period (weeks), median (IQR)		39 (23-40)	39 (36-40)
Prepregnancy BMI (kg/m^2^), median (IQR)		22.8 (15.8-32.7)	21.5 (17.6-30)
**Parity**			
	Unipara, n (%)	19 (63)	18 (60)
	Multipara, n (%)	11 (37)	12 (40)
History of obstetric abnormality, n (%)		3 (10)	3 (10)
**TPOAb^a^ (IU/mL), n (%)**			
	Positive	21 (70)	18 (60)
	Negative	9 (30)	12 (40)
Levothyroxine (μg/d), median (IQR)		50 (50-75)	50 (50-75)

^a^TPOAb: thyroid peroxidase antibodies.

### Primary Outcome

At baseline, the mean thyrotropin levels were 4.6 (SD 1.4) mIU/L in the standard group and 4.4 (SD 0.9) mIU/L in the metaverse group. After LT4 treatment, the mean thyrotropin level at delivery was 2.0 (SD 0.1) mIU/L in the standard group and 1.9 (SD 0.1) mIU/L in the metaverse group (*P*=.83). At 6 weeks post partum, the mean thyrotropin levels were 2.7 (SD 0.1) mIU/L in both groups (*P*=.78).

Maternal adverse outcomes occurred in 43% (13/30) of the standard group and 37% (11/30) of the metaverse group (*P*=.60). These included gestational diabetes, preeclampsia, premature rupture of membranes, and amniotic fluid abnormalities (Table 2). Offspring baseline characteristics showed no significant differences between the two groups (Table S1 in Multimedia Appendix 1). The median birthweight was 3290 (IQR 1830-3940) g in the standard group and 3315 (IQR 2870-3660) g in the metaverse group (*P*=.91). Two newborns in the standard group were diagnosed as small for gestational age. Adverse offspring outcomes were significantly lower in the metaverse group (2/30, 7%) compared to the standard group (10/30, 33%; *P*=.01). These outcomes included preterm birth, fetal distress, neonatal intensive care unit admissions, and neonatal hypoxemia (Table 3).

**Table 2:** Adverse outcomes of maternity.

	Standard group (n=30), n (%)	Metaverse group (n=30), n (%)	*P* value
Gestational diabetes mellitus	6 (20)	4 (13)	.49^a^
Hypertensive disorder complicating pregnancy	0 (0)	0 (0)	>.99^b^
Severe preeclampsia	1 (3)	0 (0)	>.99^b^
Threatened abortion	0 (0)	1 (3)	>.99^b^
Premature rupture of fetal membranes	5 (17)	5 (17)	.25^a^
Abnormal amniotic fluid	1 (3)	1 (3)	>.99^b^
Postpartum hemorrhage	0 (0)	0 (0)	>.99^b^
Total incidence	13 (43)	11 (37)	.60^a^

^a^Pearson *χ*^2^ test.

^b^Continuity correction test.

**Table 3:** Adverse outcomes of the offspring.

	Standard group (n=30)^a^, n (%)	Metaverse group (n=30), n (%)	*P* value
Abnormal perinatal ultrasound and fetal heart monitoring	0 (0)	0 (0)	>.99^b^
Low perinatal weight	1 (3)	0 (0)	>.99^b^
Preterm birth	3 (10)	1 (3)	.61^b^
Stillbirth	0 (0)	0 (0)	>.99^b^
Miscarriage	1 (3)	0 (0)	>.99^b^
Neonatal death	0 (0)	0 (0)	>.99^b^
Admission to neonatal intensive care unit	1 (3)	0 (0)	>.99^b^
Antepartum fetal distress	2 (7)	1 (3)	>.99^b^
Fetal asphyxia^c^	1 (3)	0 (0)	>.99^b^
Fetal malformation	0 (0)	0 (0)	>.99^b^
Neonatal hypoxemia	1 (3)	0 (0)	>.99^b^
Neonatal hypoxic-ischemic encephalopathy	0 (0)	0 (0)	>.99^b^
Neonatal pneumonia or wet lung	0 (0)	0 (0)	>.99^b^
Neonatal jaundice	0 (0)	0 (0)	>.99^b^
Neonatal hypothyroidism	0 (0)	0 (0)	>.99^b^
Total incidence	10 (33)	2 (7)	.01^d^

^a^One pregnant woman in the standard group had a termination due to fetal growth restriction.

^b^Continuity correction test.

^c^Fetal asphyxia refers to an Apgar score <4 at 1 minute or an Apgar score <7 at 5 minutes.

^d^Pearson *χ*^2^ test.

Neonatal thyroid function showed no significant differences between the groups at birth, 14 days post partum, and 28 days post partum (Tables S2-S4 in Multimedia Appendix 1). The median thyrotropin levels at birth were 7.0 (IQR 3.2-16.2) mIU/L in the standard group and 6.2 (IQR 3.0-16.4) mIU/L in the metaverse group (*P*=.31). By 14 days post partum, the median thyrotropin level had decreased to 5.3 (IQR 3.5-7.8) mIU/L in the standard group and 5.0 (IQR 3.2-5.7) mIU/L in the metaverse group (*P*=.26). At 28 days post partum, median thyrotropin levels were 4.8 (IQR 3.2-6.1) mIU/L in the standard group and 4.7 (IQR 3.2-5.4) mIU/L in the metaverse group (*P*=.68). FT4 levels also showed no significant differences between the groups at any time point (all *P*>.05).

### Secondary Outcomes

At 1 month of age, neurobehavioral scores showed no significant differences between the groups across all domains (Table 4). The median scores for the standard group in gross motor, fine motor, adaptability, language, and social-emotional domains were 99 (IQR 85.5-109.8), 100 (IQR 83.3-109), 99 (IQR 86-107.3), 101.25 (IQR 85-106.8), and 101 (IQR 87.8-107), respectively. The corresponding scores for the metaverse group were 100 (IQR 90.8-109), 100.9 (IQR 93-108), 100 (IQR 95-109.0), 102 (IQR 94-107.8), and 100.5 (IQR 92-109), respectively. At 3 months of age, similar results were observed, with neurobehavioral scores remaining comparable between groups (Table 4). The median scores for the standard group were 100 (IQR 88-111) for gross motor, 101 (IQR 89-113.3) for fine motor, 102.8 (IQR 88-112.8) for adaptability, 103.7 (IQR 88.8-111.3) for language, and 104.0 (IQR 90.8-111) for social-emotional domains. Scores for the metaverse group were 102 (IQR 92.8-109.8), 103.0 (IQR 95-112.8), 104 (IQR 96-114), 104.4 (IQR 96-111.0), and 104.9 (IQR 92.8-110), respectively.

Maternal mental health outcomes were assessed via the SAS and SDS. The baseline of the mean SAS scores was 44.1 (SD 8.3) in the standard group and 43.8 (SD 7.9) in the metaverse group. It showed significantly lower scores in the metaverse group compared to the standard group at delivery (SAS: mean 44.9, SD 1.0 vs mean 50.0, SD 1.0; *P*=.001; SDS: mean 44.7, SD 0.9 vs mean 49.7, SD 0.9; *P*<.001). This trend persisted at 6 weeks post partum, with the metaverse group continuing to exhibit lower scores (SAS: mean 46.9, SD 1.0 vs mean 51.3, SD 1.0; *P*=.002; SDS: mean 47.9, SD 1.0 vs mean 51.6, SD 1.0; *P*=.009).

**Table 4:** Comparison of neuropsychological development by the Gesell Development Scale.

		Standard group scores (n=30), median (IQR)^a^	Metaverse group scores (n=30), median (IQR)	*P* value^b^
**At 1 month old**				
	Gross motor	99 (85.5-109.8)	100 (90.8-109)	.24
	Fine motor	100 (83.3-109)	100.9 (93-108)	.61
	Adaptability	99 (86-107.3)	100 (95-109.0)	.06
	Language	101.3 (85-106.8)	102 (94-107.8)	.17
	Social-emotional response	101 (87.8-107)	100.5 (92-109)	.64
**At 3 months old**				
	Gross motor	100 (88-111)	102 (92.8-109.8)	.48
	Fine motor	101 (89-113.3)	103.0 (95-112.8)	.61
	Adaptability	102.8 (88-112.8)	104 (96-114)	.17
	Language	103.7 (88.8-111.3)	104.4 (96-111.0)	.31
	Social-emotional response	104.0 (90.8-111)	104.9 (92.8-110)	.16

^a^One pregnant woman in the standard group had a termination due to fetal growth restriction.

^b^Wilcoxon rank sum test.

## Discussion

The post–COVID-19 era has underscored the transformative potential of Health 4.0, which integrates advanced technologies such as the metaverse, blockchain, and intelligent algorithms into health care systems [20]. The metaverse presents expansive opportunities, including avatar-based consultations for remote diagnoses, immersive simulations for medical training, and social interactions to enhance patient engagement [21]. Despite its growing application in various medical fields, its use in managing thyroid disorders, particularly during pregnancy, remains underexplored [22]. Thyroid dysfunction during pregnancy, especially SCH, has gained increasing attention due to its often overlooked association with adverse pregnancy outcomes [23]. The adoption of innovative technologies like the metaverse to provide personalized care for pregnant women with SCH is crucial for improving maternal and neonatal health.

Recent studies have focused on the etiology, diagnosis, and management of adverse pregnancy outcomes [23,24]. Mounting evidence links SCH during pregnancy to elevated risks of complications. For instance, a meta-analysis reported a higher incidence of preterm birth in women with SCH (odds ratio 2.9, 95% CI 1.0-1.6) [25]. Similarly, a retrospective study found that SCH was associated with increased risks of preterm birth (relative risk 2.2, 95% CI 1.1-4.0) and neonatal respiratory distress syndrome (relative risk 2.8, 95% CI 1.0-7.8), particularly in early pregnancy [26]. These findings emphasize the importance of timely and effective interventions to improve outcomes [17]. Inspired by the establishment of virtual home hospitals in the United States in 2019, our study used virtual simulation technologies for remote monitoring, enabling timely evaluations and interventions [27]. Notably, the incidence of adverse offspring outcomes was significantly lower in the metaverse group compared to the standard group. Although maternal adverse outcomes did not differ significantly between groups (13/30, 43% vs 11/30, 37%), a favorable trend was observed. Neurodevelopmental outcomes, assessed using GDS scores, showed no significant differences between groups. To our knowledge, this is the first study to explore the application of a metaverse-based platform for the management of pregnant women with SCH. Regular treatment and continuous monitoring are essential for this population, and the dynamic follow-up capabilities of the metaverse platform are particularly well suited to their needs. The platform facilitates remote consultations, real-time monitoring, and comprehensive patient education through individualized treatment plans [24-26,28]. Compared to conventional health care services, the metaverse platform offers a more immersive and accessible experience, which may improve engagement and adherence to care protocols.

**Figure 3:**
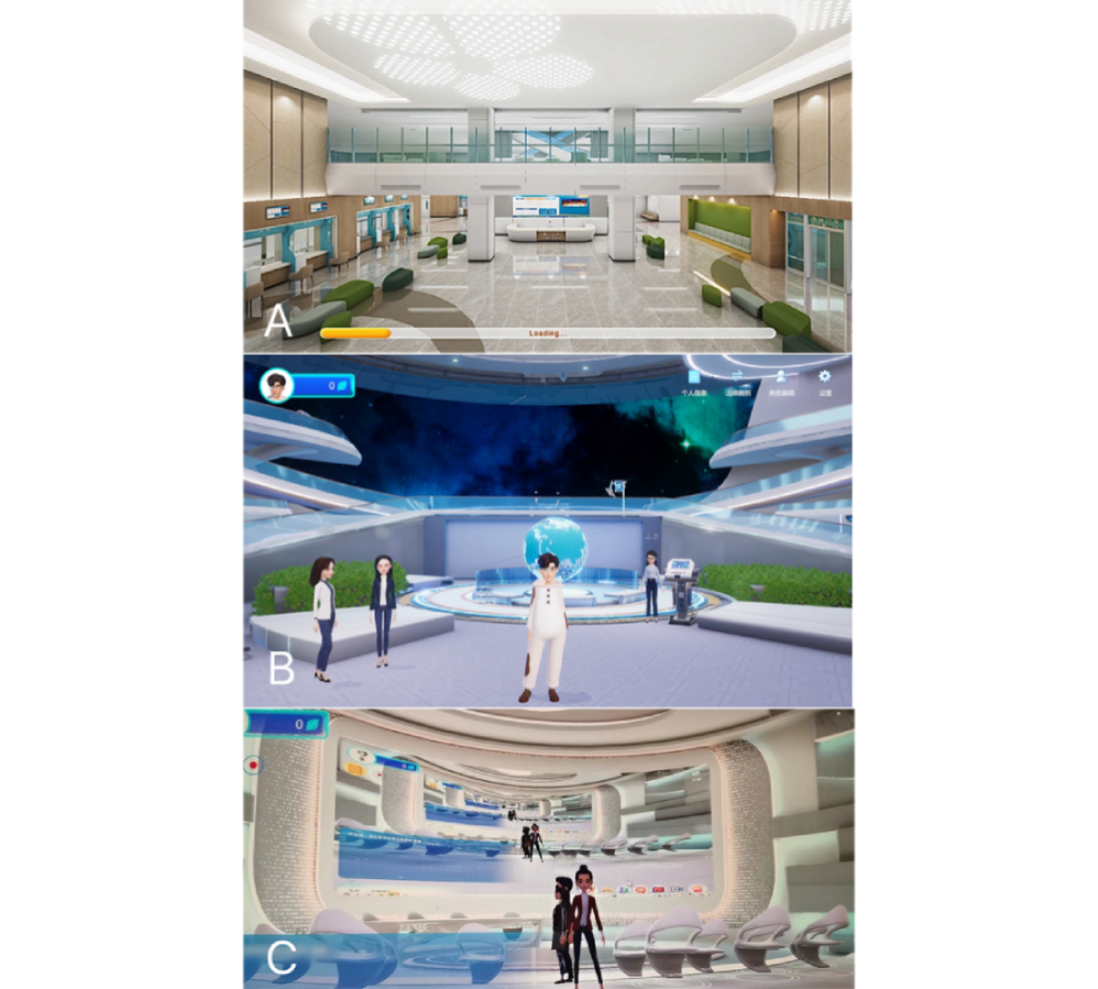
Screenshots from metaverse clinic, a virtual clinic. (A) Clinic scene. (B) Clinic chatting room. (C) Clinic conference room.

As illustrated in Figure 3, a virtual clinic was developed to simulate a traditional outpatient environment. Both investigators and participants created digital avatars, enabling seamless access to the virtual clinic from any location. This setup allowed participants to communicate directly with investigators, sharing critical information on medication dosages, test results, diet, lifestyle, and emotional well-being. By eliminating the need for frequent hospital visits, this model fostered greater engagement in the treatment process. Moreover, the platform addressed health care disparities by delivering specialized care to patients in remote or underserved areas.

**Figure 4:**
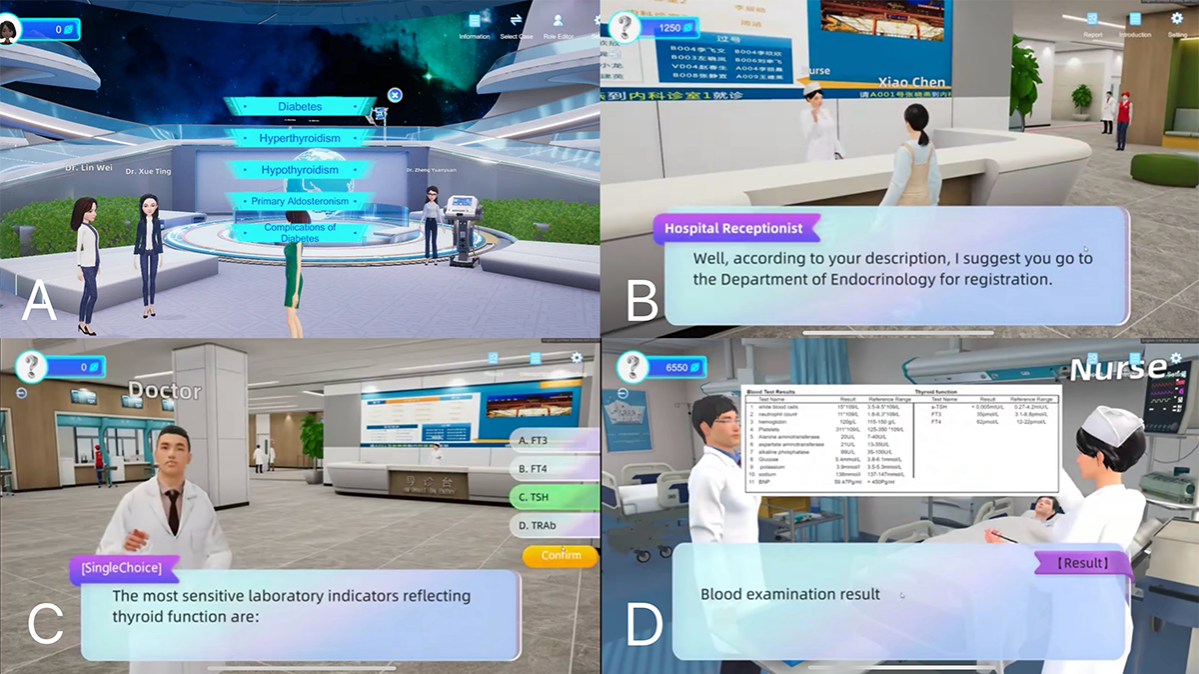
Screenshots from the metaverse medical education games. (A) Metaverse medical games option. (B) Metaverse hospital receptionist. (C) Stage mode of metaverse medical games. (D) Inpatient ward scene in metaverse medical game.

Maternal mental health is a vital component of perinatal care. Studies indicate that approximately one-quarter of pregnant women with SCH are diagnosed with depression [29]. Impaired mental health during the perinatal period has been linked to adverse outcomes for both mothers and their offspring, including preterm birth, miscarriage, impaired fetal development, and an increased risk of autism spectrum disorders [30-33]. Improving the emotional well-being of perinatal mothers has therefore become a focus of recent research aimed at enhancing pregnancy outcomes [34,35]. Our study explored the use of a metaverse platform to address emotional disorders in pregnant women with SCH. Evidence supports the efficacy of VR in managing anxiety, depression, and fear [36]. Additionally, metaverse platforms have demonstrated success in providing nonpharmacological interventions for conditions such as posttraumatic stress disorder, depression, and cognitive decline [37]. These platforms enable the creation of highly customized therapeutic environments tailored to individual needs and treatment goals. The incorporation of gamification and interactive scenarios further enhances patient engagement, improving adherence to treatment plans [38,39] (Figure 4). Compared to the standard group, patients in the metaverse group showed significant improvements in emotional well-being and a lower incidence of adverse offspring outcomes, consistent with previous studies. These findings underscore the positive impact of addressing maternal emotional health on offspring outcomes. However, pregnancy is a complex process, and further mechanistic studies are necessary to elucidate the pathways underlying these effects. Our study suggests that the metaverse platform has the potential to benefit all pregnant women, not just those with SCH. By alleviating anxiety and providing foundational medical knowledge, such platforms could enhance maternal well-being and perinatal outcomes on a broader scale.

This study has several notable strengths. To our knowledge, it is the first to use a metaverse platform for managing pregnant women with SCH. Compared to traditional telemedicine services, the metaverse offers advancements, particularly in safeguarding patient privacy. The secure virtual environment minimizes data leakage risks, alleviating psychological burdens and reducing the embarrassment often associated with medical consultations. The anonymity afforded by virtual interactions encourages patients to communicate more openly and confidently with physicians, enhancing both diagnostic and therapeutic efficacy. Furthermore, the study extends beyond pregnancy-related adverse outcomes to include assessments of psychological well-being. The metaverse platform also demonstrates the potential to address global disparities in medical resources by enabling equitable access to high-quality health care services, regardless of geographic or economic constraints. This approach represents a step forward in fostering a more balanced distribution of medical resources and advancing global health systems.

Despite these strengths, the study has limitations. Its single-center design and relatively small sample size may restrict the generalizability of the findings. Efforts are underway to expand the application of the metaverse platform to more diverse populations and other endocrine disorders to evaluate its broader effectiveness. Furthermore, the current platform remains in its developmental stage. Dependence on high-quality interactive devices and associated costs limited participant recruitment, potentially introducing statistical bias.

The study highlights the efficacy of an innovative metaverse-based follow-up management system for pregnant women with SCH. Compared to conventional care, this platform significantly reduced adverse offspring outcomes, achieved comparable maternal adverse outcomes, and markedly alleviated psychological distress during pregnancy. By enabling remote access to specialized and personalized care, the metaverse platform addresses disparities in medical resource distribution. In the digital era, this technology offers a transformative approach to managing endocrine disorders, particularly thyroid diseases, with the potential to enhance patient outcomes and advance equitable health care delivery.

## Appendix

Multimedia Appendix 1 Supplementary tables.

Multimedia Appendix 2 CONSORT-eHEALTH checklist (V 1.6.1).

## Abbreviations

FT4: free thyroxine
GDS: Gesell Development Scale
LT4: levothyroxine
SAS: Self-Rating Anxiety Scale
SCH: subclinical hypothyroidism
SDS: Self-Rating Depression Scale
VR: virtual reality

## Data Availability

The datasets generated or analyzed during this study are available from the corresponding author upon reasonable request.
