# Supplementary material for: Metaverse Clinic for Pregnant Women With Subclinical Hypothyroidism: Prospective Randomized Study
Source: J Med Internet Res. 2025 Feb 5;27:e64634. doi: 10.2196/64634 (PMC11840391; doi:10.2196/64634)
Supplement: Multimedia Appendix 1 [file jmir_v27i1e64634_app1.docx]

**Table S1: Baseline characteristics of the offspring.**

|  | **The standard group (*n*=30)** ^a^ | **The Metaverse group (*n*=30)** | ***P*** ^b^ |
| --- | --- | --- | --- |
| Birthweight, grams,  Median (IQR) | 3290 (1830-3940) | 3315 (2870-3660) | 0.909 |
| Sex, n |  |  |  |
| Male/Female | 17/12 | 13/17 | 0.240 |
| SGA ^c^, n |  |  |  |
| Yes/No | 2/29 | 0/30 | 1 |
| LGA ^d^, n |  |  |  |
| Yes/No | 0/29 | 0/30 | 1 |

a: One pregnant woman in the standard group had a termination due to fetal growth restriction.

b: The Wilcoxon rank-sum test, Pearson Chi-squared test or Continuity correction test.

c: SGA, small for gestational age.

d: LGA, large for gestational age.

**Table S2: Thyroid function test of offspring at birth.**

|  | At birth | | *P* ^b^ |
| --- | --- | --- | --- |
|  | The standard group  (*n*=30) ^a^ | The Metaverse group  (*n*=30) |  |
| TSH ^c^ (mIU/L),  Median (IQR) | 7 (3.2-16.2) | 6.2 (3.0-16.4) | 0.309 |
| <0.7, n (%) | 0 (0) | 0 (0) |  |
| <6, n (%) | 11 (36.7) | 14 (46.7) |  |
| 6-20, n (%) | 18 (60) | 16 (53.3) |  |
| >20, n (%) | 0 (0) | 0 (0) |  |
| >40, n (%) | 0 (0) | 0 (0) | 0.497 |
| FT4 ^d^ (pmol/L),  Median (IQR) | 27 (22.9-34.9) | 26.7 (19-31.8) | 0.467 |
| <11, n (%) | 0 (0) | 0 (0) |  |
| 11-32, n (%) | 27 (90) | 30 (100) |  |
| >32, n (%) | 2 (6.7) | 0 (0) | 0.457 |

a: One pregnant woman in the standard group had a termination due to fetal growth restriction.

b: The Wilcoxon rank-sum test, Pearson Chi-squared test or Continuity correction test

c: TSH: Thyroid stimulating hormone.

d: FT4: Free thyroxine.

**Table S3: Thyroid function test of offspring at 14 days post partum.**

| **Outcomes** | **The standard group**  **(*n*=30)** ^a^ | **The Metaverse group**  **(*n*=30)** | ***P*** ^b^ |
| --- | --- | --- | --- |
| TSH ^c^ (mIU/L),  Median (IQR) | 5.3  (3.5-7.8) | 5.02  (3.2-5.7) | 0.261 |
| <0.7, n (%) | 0 (0) | 0 (0) | 1 |
| <6, n (%) | 28 (93.3) | 30 (100) | 1 |
| 6-20, n (%) | 1 (3.3) | 0 0) | 1 |
| >20, n (%) | 0 (0) | 0 (0) | 1 |
| >40, n (%) | 0 (0) | 0 (0) | 1 |
| FT4 ^d^ (pmol/L)  Median (IQR) | 23.2  (19-27.0) | 23.4  (19-27) | 0.721 |
| <11.5, n (%) | 0 (0) | 0 (0) | 1 |
| 11.5-28.3, n (%) | 29 (96.7) | 30 (100) | 1 |
| >28.3, n (%) | 0 (0) | 0 (0) | 1 |

a: One pregnant woman in the standard group had a termination due to fetal growth restriction.

b: The Wilcoxon rank-sum test, Pearson Chi-squared test or Continuity correction test

c: TSH: Thyroid stimulating hormone.

d: FT4: Free thyroxine.

**Table S4: Thyroid function test of newborn at 28 days post partum.**

| **Outcomes** | **The standard group**  **(*n*=30)** ^a^ | **The Metaverse group**  **(*n*=30)** | ***P*** ^b^ |
| --- | --- | --- | --- |
| TSH ^c^ (mIU/L),  Median (IQR) | 4.8  (3.2-6.1) | 4.7  (3.2-5.4) | 0.682 |
| <0.7, n (%) | 0 (0) | 0 (0) | 1 |
| <6, n (%) | 29 (96.7) | 30 (100) | 1 |
| 6-20, n (%) | 0 (0) | 0 (0) | 1 |
| >20, n (%) | 0 (0) | 0 (0) | 1 |
| >40, n (%) | 0 (0) | 0 (0) | 1 |
| FT4 ^d^ (pmol/L),  Median (IQR) | 22  (18.8-24.9) | 21.68  (18.8-25.0) | 0.897 |
| <11.5, n (%) | 0 (0) | 0 (0) | 1 |
| 11.5-28.3, n (%) | 29 (96.7) | 30 (100) | 1 |
| >28.3, n (%) | 0 (0) | 0 (0) | 1 |

a: One pregnant woman in the standard group had a termination due to fetal growth restriction.

b: The Wilcoxon rank-sum test, Pearson Chi-squared test or Continuity correction test.

c: TSH: Thyroid stimulating hormone.

d: FT4: Free thyroxine.
